# Formal comment on: Parent reports of adolescents and young adults perceived to show signs of a rapid onset of gender dysphoria

**DOI:** 10.1371/journal.pone.0212578

**Published:** 2019-03-19

**Authors:** Angelo Brandelli Costa

**Affiliations:** 1 Psychology Graduate Program, Pontifical Catholic University of Rio Grande do Sul, Porto Alegre, Brazil; 2 Gender Identity Program, Hospital de Clínicas de Porto Alegre, Porto Alegre, Brazil; University of Pennsylvania, UNITED STATES

I have read with great attention the article by Lisa Littman published in *PLOS ONE* [1]. Further study on the forms of presentation of gender dysphoria in childhood and adolescence is imperative since we still lack consensus regarding the best diagnostic and treatment approaches for this matter. Dr. Littman’s main objectives were “to (1) collect data about parents’ observations, experiences, and perspectives about their AYA children showing signs of a rapid onset of gender dysphoria that began during or after puberty, and (2) develop hypotheses about factors that may contribute to the onset and/or expression of gender dysphoria among this demographic group” [1].

One possibility to address the purpose that the study originally proposes is to follow a group of gender variant young people evaluated by mental health professionals in a longitudinal way, to assess if those who persist demanding gender affirmation differ (in terms of contact and social influence, or other factors) from those who do not persist. Another (much simpler) approach could involve a cross-sectional design, in which transgender youth answered questions concerning their networks and peer influence. In contrast to those possible approaches, Dr. Littman’s research provides only indirect evidence of the role of the influence of social and media contagion on young people’s gender identity. Littman's article recruited parents online. Some of the websites that posted recruitment information about the study might attract parents who are more likely to question their child's gender self-identification and the current best healthcare approaches. No youth were enrolled.

Several studies have pointed out the importance of involving young people in studies of their health [2]. From a bioethical point of view, despite several dilemmas [3], this need is guided by the principle of the best interest of children and their right to be represented in the matters that affect them [4]. In this regard, with respect to medical procedures related to gender in childhood (in trans and intersex cases), the WHO among other agencies [5], already recognized the need to take children’s voices into account in order to avoid coercive treatments: "the best interests of the child should always be the primary concern, giving due weight to the views of children in accordance with their age and maturity, and taking into account their evolving capacity for decision-making" (p13).

Evidence also points to a low correlation between parents' and children’ self-evaluation in several domains of mental health [6]. For example, regarding quality of life, a systematic review verified that parent and children do not agree in the evaluation for children non-observable states (such as emotions) [6]. The authors point to the need for collecting information from both parts. The same seems to be true in the assessment of children’s anxiety [7]. This discrepancy may be due to parental attribution bias in the recollection of children's medical history [8]. Furthermore, parents’ biases may be enhanced in the presence of stress [9] and psychological symptoms [10]. Studies have shown that this could be the case for a good proportion of parents of gender-variant children and adolescents, who tend to present negative attitudes toward their offspring gender variation [11, 12].

The level of evidence produced by the Dr. Littman’s study cannot generate a new diagnostic criterion relative to the time of presentation of the demands of medical and social gender affirmation. Several procedures still need to be adopted to generate a potential new subcategory of gender dysphoria that has not yet been clinically validated. One of these procedures is the assessment of mental health professionals trained according to the World Professional Association for Transgender Health (WPATH) [13] and the American Psychological Association (APA) [14] guidelines, interviewing not just the family, but the youth (longitudinally).

In addition, it is important to note that psychological distress, which is investigated as an outcome in the study in question, is not central to the new diagnosis of gender incongruity proposed by WHO in the new International Classification of Diseases, ICD-11. WHO removed transsexualism from the chapter of psychiatric conditions in the ICD-10 and placed gender incongruence in a chapter of general sexual health and recognizing that the psychological distress could be the result of stigmatization and maltreatment, rather than an intrinsic aspect of gender identity [15].

Parental anxiety seems to increase with the level of gender nonconformity of their children and this anxiety is associated with negative impacts on the well-being of their children [16]. It is therefore not surprising that growing up without proper healthcare and in families that do not support gender and sexual diversity may negatively impact the mental health outcomes of gender variant young people (growing-up to be trans-adults or not) [17].

In this regard, it should be noted that not all children with gender variability grow to be transgender adults and that a transgender adult does not always grow from a childhood diagnosis [18]. In the WPATH’ Standards of Care for the Health of Transsexual, Transgender, and Gender Nonconforming People [13], one of the roles of mental health professionals working with children and adolescents with gender dysphoria is to help families to have an accepting and nurturing response to the concerns of their children. Families should be supported in managing uncertainty and anxiety; thus, helping youth to develop a positive self-concept. This does not necessarily mean consenting with an early transition. The American Psychological Association has categorically stated that healthcare professionals should be encouraged to educate themselves about the advantages and disadvantages of social transition during childhood and adolescence and discuss these factors with their youth clients and their parents. It is fundamental to emphasize to parents the importance of allowing their children to be free to return to a gender identity that is aligned with the sex assigned at birth at any point, if it is the case [14].

These developmental complexities are often neglected and deserve further investigation. Data such as those collected by Dr. Littman about parents' views and experiences with youth who show sudden signs of gender dysphoria should be further investigated and documented. The forms of presentation of gender variations in childhood are little known, the clinical management of these children is not fully established, and the refinement of the diagnostic criteria are imperative. However, we must always keep in mind the role that transphobia (still prevalent [19]) has in the negative impact that this gender variation has on society, parents, and therefore on children.
